# Erratum: Review on Approved and Inprogress COVID-19 Vaccines

**DOI:** 10.5812/ijpr-146470

**Published:** 2024-02-27

**Authors:** Soraya Shahhosseini

**Affiliations:** 1 School of Pharmacy, Shahid Beheshti University of Medical Sciences, Tehran, Iran

In the article titled 'Review on Approved and in-progress COVID-19 Vaccines,' published in Iran J Pharm Res. 2022; 21(1): e124228, there was an error in the authorship. The correct name of the third author is Vahideh Montazeri.

